# Sustainable valorization of artisanal sour buttermilk using nanofiltration and spray drying

**DOI:** 10.3389/fnut.2026.1790618

**Published:** 2026-05-27

**Authors:** Subhadip Manik, Ganga Sahay Meena, Yogesh Khetra, Ashish Kumar Singh, Sumit Arora, Richa Singh, Raghu H. V.

**Affiliations:** 1 Dairy Technology Division, ICAR-National Dairy Research Institute, Karnal, Haryana, India; 2 Dairy Chemistry Division, ICAR-National Dairy Research Institute, Karnal, Haryana, India; 3 Dairy Microbiology Division, ICAR-National Dairy Research Institute, Karnal, Haryana, India

**Keywords:** coagulant, conservation, nanofiltration, sour buttermilk, spray drying, waste

## Abstract

Sour buttermilk is formed when milk fat is conserved as clarified fat using a traditional household method. It has low total solids (TS) and high acidity. Often, people discard sour buttermilk due to a lack of an effective preservation method. This investigation aimed to concentrate sour buttermilk using nanofiltration (NF) equipped with a 200 Da molecular weight cut-off polyamide membrane (AFC 40), tubular B1 parallel flow module, operated at 20 ± 1 bar pressure and 35 ± 1 °C temperature. The process yielded an NF retentate with 19.61% total solids. The retentate was then spray dried at 185/75 ± 5 °C to produce sour buttermilk powder. This powder showed excellent wettability (3 s, instant) and flowability. The NF permeate contained 0.42 ± 0.05% TS, 0.12 ± 0.03% ash, 0.37 ± 0.02% lactic acidity, and 1.79 ± 0.21 mPa.s apparent viscosity at 20 °C. It was used as a natural coagulant to prepare heat and acid-coagulated milk gel (*chhana*) from buffalo milk. Usually, buffalo milk *chhana* has a hard texture and grainy body. When prepared with NF permeate, buffalo milk *chhana* had significantly (*p* < 0.05) lower hardness and higher yield compared to cow milk *chhana* made with citric acid. Overall, this investigation showed that NF and spray drying can sustainably valorize sour buttermilk with zero waste generation.

## Graphical abstract

**Figure:**
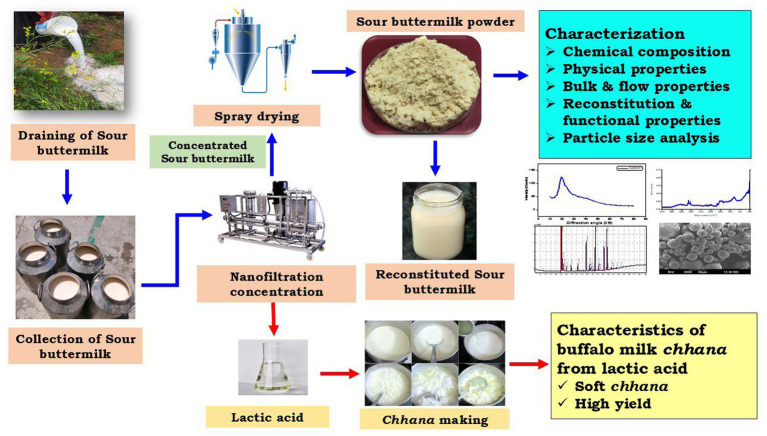

## Highlights

- Nanofiltration efficiently enhanced total solids of sour buttermilk
- Nanofiltration permeate was directly used as a natural coagulant
- A sustainable method was developed to conserve milk solids in sour buttermilk

## 1 Introduction

Food loss and waste have become significant barriers to sustainability in recent times due to their impact not only on the environment, climate, and resources (1–4), but also on the economy (5–7) and nutrition (8–10). It is estimated that one-third of the global food produced for human consumption is lost or wasted annually, which costs around USD 1 trillion (11–13). The dairy processing sector significantly contributes to environmental pollution via global production of 4–11 million tonnes of solid waste and effluents per year (14, 15). Additionally, an effluent ratio of 1–3 times was produced per liter of milk processed, contributing 3.739–11.217 million cubic meters of waste annually (14, 16).

With 25% contribution, India is a global leader in milk production with 231 MT during 2022–2023 (17). Of the total Indian milk production, 14% is converted into fermented products in the unorganized sector (18). In the countryside, farmers artisanally processed surplus milk by an indigenous method consisting of heating, cooling, fermentation, and churning to produce clarified fat (*ghee*), which preserved the milk fat. During the production of 1 Kg of *ghee*, 15–20 Kg of artisanal sour buttermilk (SBM) is generated (19, 20). SBM contains 3.8% of total solids (TS), 1.29% of protein, 0.8% of fat, 1.2% of lactose, 0.4% of ash, and 0.44% of lactic acid, respectively (21). It has a brownish color arising from the prolonged heat treatment [75–95 °C/1–10 h; (22)] of milk prior to its inoculation with an unspecified starter culture that results in uncontrolled fermentation. It contains numerous curd particles and a non-homogeneous consistency, which is prone to deposition of curd material and watery portion on the top because of its higher acidity (22, 23). Its short shelf life, high acidity, poor heat stability, large volume, and inadequate collection and processing systems are the major issues that have made its utilization challenging so far (24). Therefore, SBM conservation has not been attempted for these reasons, and it is directly discharged into the environment.

Nanofiltration (NF) and Reverse Osmosis (RO) are well-known pressure-driven membrane processes that have shown strong potential for treating dairy wastewater. These processes are capable of retaining fat, protein, and lactose and lowering chemical oxygen demand (COD) in the resulting permeate (25–28). Numerous studies advocate the use of RO and NF for treating dairy wastewater (29–36). Additionally, NF effectively separated lactic acid (~100%) into the permeate from fermented cheese whey (37). As per Bobrova and Ostretsova (38), NF successfully achieved a 2.48-fold concentration of sweet cream buttermilk (SCBM) from 8.10–20.10% TS. Zscherpe et al. (39) concentrated pasteurized bovine skim milk up to 40% of dry matter using an RO-NF membrane cascade. NF was found to be advantageous at high skim milk concentrations compared to RO because monovalent ions were passed into the NF permeate, thereby decreasing the osmotic pressure of the retentate and affecting the filtration process (40). This facilitates the higher concentration of lactose and proteins in the retentate.

Spray drying is a well-established, rapid, and cost-effective method. It reduces bulk and transportation costs and increases product stability by lowering water activity, simplifying application in various food formulations (41). Previously, authors concentrated on SBM (4.13% TS) using RO, but the concentration was restricted to 12.98% TS. The resulting retentate was then spray-dried to produce sour buttermilk powder (SBMP). Since RO had limitations in SBM concentration, the current investigation evaluates the suitability of NF for SBM concentration. Additionally, the generated permeate, containing lactic acid, was used as a natural coagulant for producing heat and acid-coagulated milk gel (chhana) from buffalo milk. Traditionally, chhana from buffalo milk is criticized for its hard body and chewy texture, making it less suitable for sweetmeats compared to chhana made from cow milk (21, 42–44). Therefore, this investigation focuses on the complete utilization of NF retentate and permeate to ensure sustainable valorization of SBM and support environmental sustainability in the dairy sector.

## 2 Materials and methods

Analytical grade chemicals were obtained from HiMedia Laboratories (Mumbai, India) and Sigma Aldrich (Bengaluru, India) and used in this study.

### 2.1 Procurement and processing of SBM

**Figure 1:**
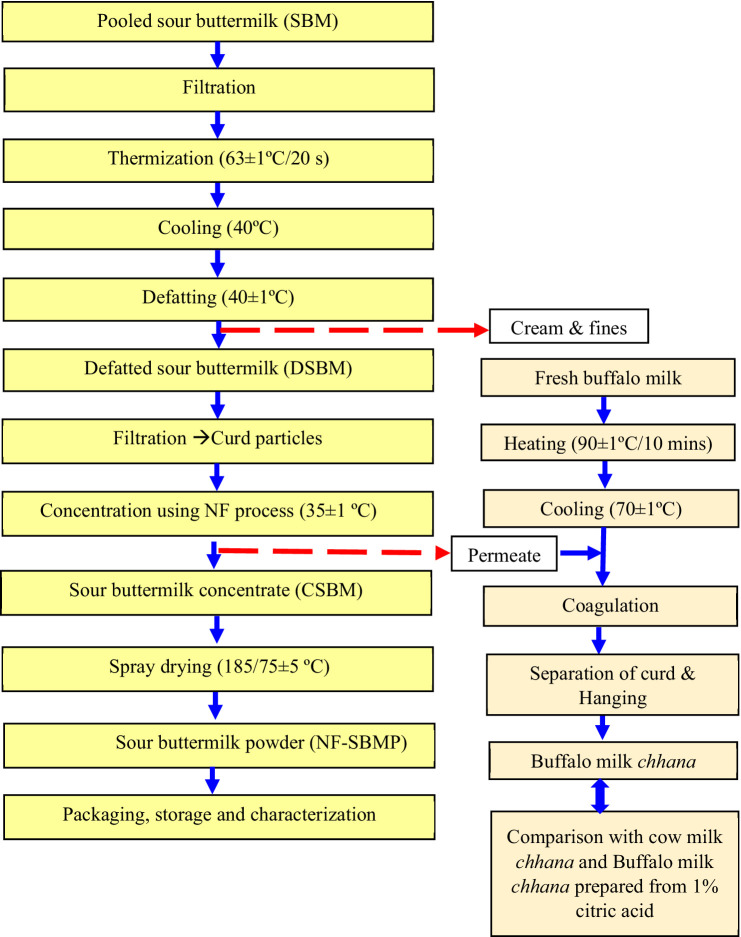
Production process of sour buttermilk powder by nanofiltration process (NF-SBMP) and buffalo milk *chhana.*

Fresh SBM samples (200 Kg) were procured from countryside farmers located in the vicinity of Karnal, India (29.6857° N, 76.9905° E) in the early morning. All samples were pooled, thermized (63 °C/ 20 s), cooled (40 ± 1 °C), and defatted using an electrical centrifuge (Model-Kamdhenu KD-600; Make: Sinhal Metal India Pvt. Ltd., New Delhi, India). Subsequently, defatted sour buttermilk (DSBM) was passed through a muslin cloth for filtration and then concentrated employing the NF process, maintaining 20 ± 1 bar trans membrane pressure (TMP) and 35 ± 1 °C temperature to obtain sour buttermilk concentrate (CSBM) with the maximum possible TS (Figure 1). The NF plant used in this investigation was procured from M/s Permionics Membranes Pvt. Ltd., Vadodara, Gujarat, India. It contains a total of 0.9 m^2^ membrane area and was equipped with a triple plunger pump (SS contact surface) to generate 60 bar pressure. The membrane (membrane material: polyamide, AFC 40; module: tubular, B1 parallel flow; length ×diameter:1.2 m × 100 mm) was procured from M/s PCI Membranes, Poland. This plant has a hold-up volume of 7 L, 200 Da molecular weight cut-off, maximum operational pressure, and temperature of 60 bar and 60 °C with a pH tolerance of 1.5–9.5.

**Figure 2:**
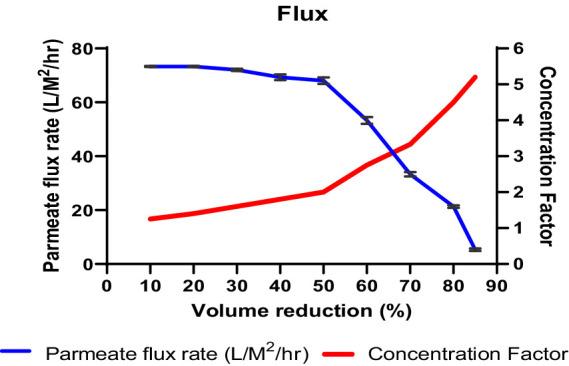
Change in permeate flux as a function of percent volume reduction ratio and concentration factor.

The change in permeate flux was recorded and represented as a function of concentration factor (CF) and percentage volume reduction ratio (VRR), as illustrated in Figure 2. The permeate was stored at refrigeration temperature (4 ± 1 °C) and further used as a natural coagulant for the preparation of *chhana* from buffalo milk as described in section 2.2 and Figure 1. The flux mean (FM) was determined using the following formula reported by St-Gelais et al. (45), which was based on initial flux (IF) and final flux (FF) values.


$$\begin{array}{l} L.m^{-2}\;h^{-1}\;\;\mathrm{FM}=\mathrm{FF}+[\;0.33\times (\text{IF}-\mathrm{FF})]=5.33\\ +[0.33\times (73.33-5.33)]=27.77\; \end{array}$$

CSBM thus produced was subsequently subjected to spray drying (185/75 ± 5 °C) using a single-stage spray drier (Jektron Pvt. Ltd., Pune, India; rotary atomizer). Metalized polyester-LDPE laminates were used to pack and store the produced sour buttermilk powder (SBMP) at 4 ± 1 °C until its further characterization.

#### 2.1.1 Chemical composition and physical properties of SBM, DSBM, CSBM, and SBMP

TS, fat, protein, and ash contents of SBM, DSBM, CSBM, and SBMP were estimated as per the protocol of AOAC (46) (925.23 and 927.05 for solids in milk and milk powder, 989.05 for fat, 991.20 for protein, and 945.46 and 930.30 for ash in milk and milk powder). Their lactose content was estimated as per the Lane Eynon method (47). Free fat content of SBMP was estimated using the method described by Hall and Hedrick (48). The methods outlined by Keeney and Bassette (49) and Hegenauer et al. (50) were used to measure hydroxymethylfurfural (HMF) and 2-thiobarbituric acid (TBA) of SBMP, respectively. pH of SBM, DSBM, CSBM, and reconstituted SBMP (10%, w/v) solutions were recorded using a pre-calibrated pH meter, while their acidity was determined as per the protocol of IS: 11766 (51). Zetasizer Nano ZS (Malvern, United Kingdom) was used for the measurement of the *ζ*-potential of these samples at 25 °C, adopting the procedure outlined by Mahadev and Meena (52), while Hunter Lab model color Flex^®^ (Mini-Scan XE plus, Hunter Associates Laboratory Inc., Reston, VA, United States) was used to record their color values. Water activity (a_w_) for SBMP was measured using Aqua Lab (Model Series 3 TE; supplied by M/s Decagon Devices, Pullman, WA, United States).

Apparent viscosity (ƞ_Appa_. mPa.s) of SBM, DSBM, CSBM, and 10% (w/v) of reconstituted SBMP solution were determined at 20 °C and 1–500 s^−1^ shear rate using a rheometer (model: MCR52; make: Anton Paar, Graz, Austria) as outlined by Manik et al. (53).

#### 2.1.2 Properties (bulk, flow, reconstitution, and functional), fatty acid profile, and morphological characteristics of SBMP

Different bulk, flow, reconstitution, and functional properties such as Interstitial air content (IAC), occluded air content (OAC), loose bulk density (LBD), packed bulk density (PBD), particle density (PD), porosity, flowability, wettability, dispersibility, solubility index, water binding capacity (WBC), oil binding capacity (OBC), foam capacity (FC), foam stability (FS), emulsion capacity (EC) emulsion stability (ES), and particle size analysis (PSA) of SBMP were estimated using standard methods as described by Manik et al. (53). Similarly, fatty acid profiling and morphological characterization of SBMP were performed as per the outlined method of Manik et al. (53).

### 2.2 Production of chhana using NF permeate as a natural coagulant

Fresh buffalo milk (6% fat, 9% SNF) was procured from Experimental Dairy, ICAR-NDRI, Karnal. A method reported by Chakraborty et al. (54) was used to prepare c*hhana*. For each trial, 10 kg of buffalo milk was heat-treated to 90 ± 1 °C, cooled at 70 ± 1 °C, and subsequently coagulated using NF permeate. A control sample was prepared using citric acid (1%, w/v solution) as shown in Figure 1. The coagulum and whey were separated using a muslin cloth. The coagulum of different *chhana* (buffalo milk citric acid-treated *chhana*-BMCAC, buffalo milk permeates treated *chhana*-BMPC) samples was hung for 10 min to remove whey and wrapped in aluminum foil for further analysis in a desiccator. Control cow milk *chhana* (to prepare positive control) was prepared using cow milk (10 kg; 6% fat, 9% SNF) and citric acid solution (1%, w/v) with the same protocol for comparison (cow milk citric acid-treated *chhana-*CMCAC).

#### 2.2.1 Analysis of chemical, physical, and textural properties of chhana samples

The moisture content of different *chhana* samples was measured according to BIS (55). The yield of different *chhana* samples was determined using the following formula reported by Chakraborty et al. (54).


$$\text{Yield}\;(\%)=(\frac{\text{Weight of chhana produced}}{\text{Weight of milk}})x100$$

The color values of different *chhana* samples were recorded similarly as mentioned in section 2.1.1. The textural attributes of different *chhana* samples were estimated using a texture profile analyzer (model: TA. HD. Plus C; make: Stable microsystem, United Kingdom) as per the described method of Bandyopadhyay et al. (56).

#### 2.2.2 Analysis of selected chemical composition and physical properties of whey streams obtained from different chhana samples

Estimation of TS, fat loss, pH, acidity, and ƞ_Appa_ of whey samples was performed using the standard described method for buttermilk samples in section 2.1.1.

### 2.3 Statistical analysis

Data obtained in this study were analyzed using one-way analysis of variance (ANOVA) in the IBM SPSS program (version 25) with *α* = 0.05. Tukey HSD was used as a *post hoc* test for comparison of means. Descriptive statistics were used to analyze SBMP parameters. GraphPad Prism software (Version 8.4) was used to produce the figures.

## 3 Results and discussion

### 3.1 Effect of the nanofiltration process on chemical composition and physical properties of different buttermilk samples

#### 3.1.1 Effect of the nanofiltration process on different buttermilk samples

The DSBM was successfully concentrated up to 5.20 × (82.68% VRR) by the NF process. Recorded value of mean flux was 27.77 L. m^−2^ h^−1^ at 20 bar and 35 °C. CF, VRR, and mean flux values obtained during DSBM concentration by RO process were 3.62×, 72%, and 15.07 L. m^−2^ h^−1^, respectively, at 35 bar and 35 °C (53). NF performed better than RO in terms of CF, VRR, and mean flux. Zscherpe et al. (39) found NF advantageous over RO during concentration of pasteurized bovine skim milk up to 40% of dry matter using the RO-NF membrane cascade. It is mainly due to the removal of monovalent ions into NF permeate, which decreases the osmotic pressure of the retentate and facilitates the higher concentration of lactose and proteins in the retentate (40). Figure 2 depicts the decline in flux rate with a rise in CF that could be attributed to the concentration polarization and fouling of the NF membrane. A similar decline in flux rate was also observed during DSBM concentration by the RO process. Brião et al. (57) recorded higher flux during concentration of dairy rinse water in the NF process over the RO process. Furthermore, NF could concentrate sweet cream buttermilk and defatted cheese whey milk up to 2.48 × and 3.29 × at 20 °C and 2.5 mPa (38).

#### 3.1.2 Change in chemical composition and physical properties of different buttermilk samples

Chemical composition and physical properties of different buttermilk samples are shown in Table 1. Padghan et al. (22) reported that the TS, protein, acidity, and pH values of SBM were 5.16, 2.26, 0.56% LA, and 3.99, respectively. As per Table 1, TS and protein content of the SBM were lower, while acidity and pH values were in line with the published data (22). Centrifugal separation of SBM led to a significant (*p* < 0.05) decrease in TS, fat, color values (L*, a*, b*), and ƞ_Appa_ of DSBM. NF concentration in DSBM significantly (p < 0.05) enhanced all chemical constituents and physical properties in CSBM relative to SBM and DSBM. Similar findings were also observed during the concentration of DSBM by the RO process. The CSBM produced by NF contained 52.48% higher TS than the CSBM obtained employing the RO process (53). Bobrova and Ostretsova (38) achieved 20.10% TS in the final retentate after concentration of sweet cream buttermilk (8.10%-initial TS) employing NF. The fat, protein, lactose, and ash content of CSBM were significantly (*p* < 0.05) higher than that of SBM and DSBM. Theoretically, 5.20 × concentration of DSBM (ash 0.37%) by ordinary evaporation or RO (0.37 × 5.20) could have resulted in 1.924% ash content in the resultant concentrate. However, the ash content of CSBM produced by the NF process was only 1.29% ash content (Table 1). Hence, 48.9% reduction in the ash content of CSBM could be attributed to the NF-based demineralization. Suárez et al. (58) also reported 27 and 36% reduction in ash content of whey and milk ultrafiltered permeate at volume concentration ratio (VCR) 4 and 4.7 in the NF process, respectively.

**Table 1:** Chemical composition and physical properties of SBM, DSBM, and CSBM samples.

Parameters		Sour buttermilk (SBM)	Defatted sour buttermilk (DSBM)	Sour buttermilk concentrate (CSBM)
TS (%)		4.08 ± 0.38^b^	3.77 ± 0.39^c^	19.61 ± 0.41^a^
Fat (%)		0.49 ± 0.09^a^	0.12 ± 0.02^b^	0.71 ± 0.06^a^
Protein (%)		1.73 ± 0.18^b^	1.76 ± 0.23^b^	9.78 ± 0.38^a^
Lactose (%)		1.46 ± 0.09^b^	1.48 ± 0.11^b^	7.64 ± 0.25^a^
Ash (%)		0.34 ± 0.04^b^	0.37 ± 0.04^b^	1.29 ± 0.08^a^
Acidity (% LA)		0.79 ± 0.03^b^	0.79 ± 0.04^b^	2.03 ± 0.08^a^
pH, 20°C		3.93 ± 0.07^b^	3.93 ± 0.03^b^	4.22 ± 0.02^a^
ζ-potential (mV) (100 × dilution), 25 °C		7.25 ± 1.29^b^	7.51 ± 4.79^b^	8.29 ± 1.70^a^
Color values	L*	81.12 ± 1.23^b^	80.97 ± 1.32^b^	83.81 ± 0.89^a^
	a*	−1.62 ± 0.40^b^	−2.10 ± 0.29^c^	0.18 ± 0.48^a^
	b*	9.41 ± 1.84^b^	8.93 ± 1.60^b^	13.52 ± 1.06^a^
Apparent viscosity (ƞ_Appa._), 100 s^−1^ (mPa.s), at 20°C		11.88 ± 2.78^b^	8.11 ± 1.69^c^	1393.50 ± 43.08^a^

Mean ± SD (*n* = 9 independent trails) with different superscripts ^abc^ is significantly different (*p* < 0.05) with each other row-wise.

**Figure 3:**
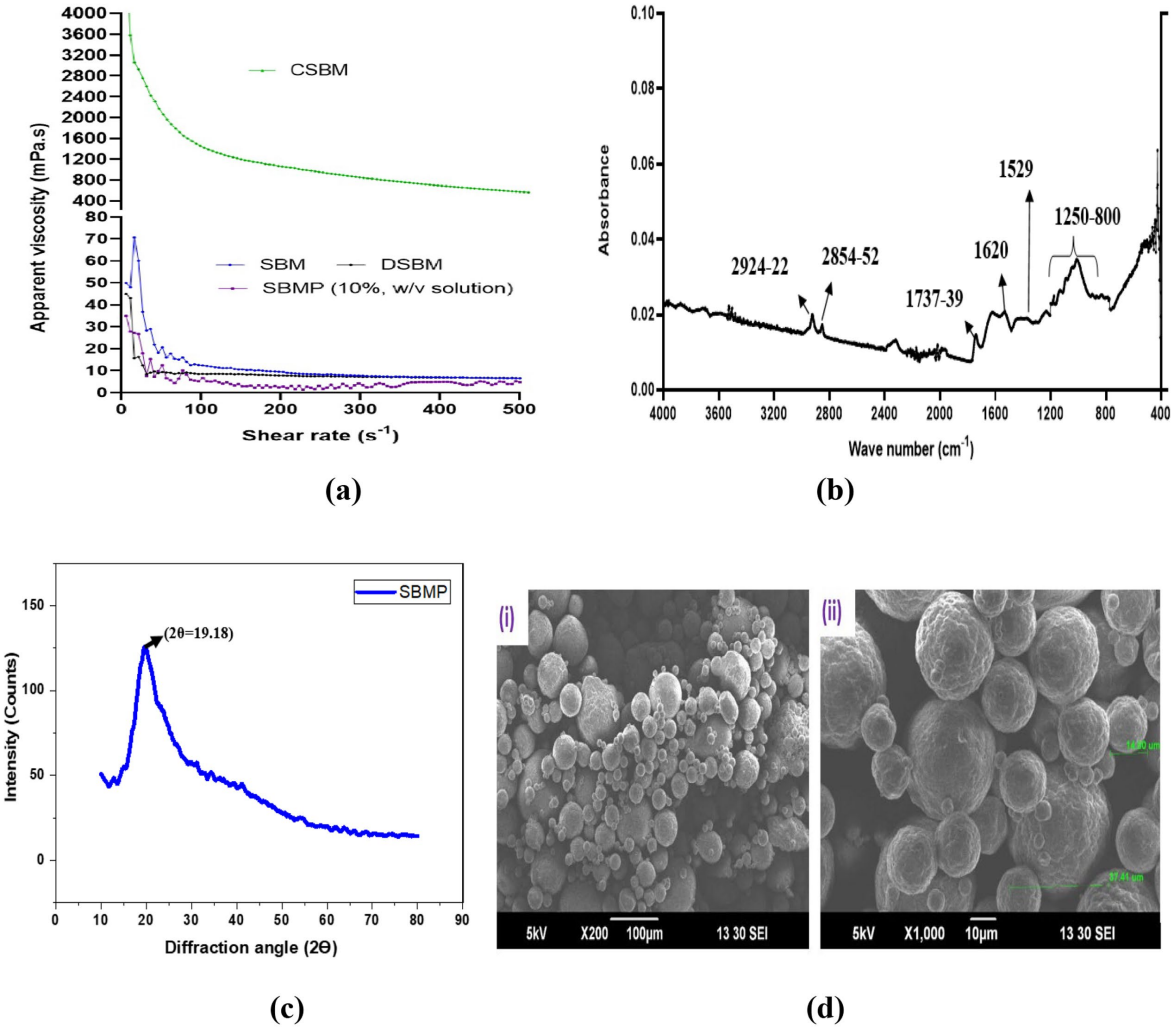
**(a)** Change in viscosity of sour buttermilk (SBM), defatted sour buttermilk (DSBM), concentrated sour buttermilk (CSBM), and reconstituted (10%, w/v) sour buttermilk powder (SBMP) samples as a function of increase in shear rate, **(b)** FTIR spectra of sour buttermilk powder (SBMP), **(c)** XRD spectra of sour buttermilk powder (SBMP), and **(d)** SEM images of sour buttermilk powder (SBMP) at (i) 200x and (ii) 1,000x magnifications.

Li et al. (37) successfully separated lactic acid and water from fermented cheese whey using the NF process. The pH of CSBM was significantly (*p* < 0.05) higher compared to DSBM, which can be easily explained by partial retention of lactic acid by the NF membrane. In comparison with DSBM, CSBM exhibited significantly (*p* < 0.05) higher *ζ*-potential, which could be attributed to its higher pH. The NF permeate obtained in this investigation had 0.42 ± 0.05% TS, 0.12 ± 0.03% ash, 0.37 ± 0.02% acidity (%LA), and 1.79 ± 0.21 mPa.s of ƞ_Appa_ at 20 °C. A significant (*p* < 0.05) increase in ƞAppa of CSBM could be explained by 5.20 × concentration of DSBM by NF process that markedly increased its TS as shown in Table 1. The change in ƞ_Appa_ values of SBM, DSBM, and CSBM as a function of change in shear rate (0–500 s^−1^) is depicted in Figure 3a.

### 3.2 Characterization of SBMP

Scientific literature pertaining to the production of SBMP is still scanty. Therefore, different properties of SBMP prepared in the current investigation were compared to the reported properties of related dairy powders, including skim milk powder (SMP), whole milk powder (WMP), *lassi* powder, yogurt powder, and buttermilk powder, whenever necessary, in support of the results. In a previous article, Manik et al. (53) produced SBMP using RO and spray drying. The SBMP powders prepared using NF and RO processes have been designated as NF-SBMP and RO-SBMP_,_ respectively. NF-SBMP was evaluated against RO-SBMP as the control for membrane-based concentration. A direct control without membrane concentration was not included, and this is recognized as a limitation. Various properties of these powders (NF-SBMP and RO-SBMP) are predominantly compared with each other. Moreover, comparisons of NF-SBMP vs. RO-SBMP are quantitative and directly comparable, whereas comparisons with other dairy powders are qualitative benchmarks used only for justification and context.

#### 3.2.1 Chemical composition and physical properties of SBMP

Table 2 shows the chemical, physical, and reconstitution properties, while Table 3 depicts the fatty acid composition of NF-SBMP. Composition of pooled SBM and process (NF or RO) applied for the concentration of DSBM caused variation in chemical makeup of NF-SBMP and RO-SBMP (53) as shown in Table 2. TS of NF-SBMP was slightly higher than that of RO-SBMP (97.82%) (Table 2), *lassi* powder [94–97%, (59)], yogurt powder [≥95%; (60)], and buttermilk powder [96–97%; (61)], respectively. The fat content of NF-SBMP was higher than that of lassi powder [1.2–2.4%; (59)] but lower than that of buttermilk powder [4.5–7.0%, (61)]. Lactose content of NF-SBMP was higher than RO-SBMP [31.34%, (53)] but lower than SMP [49.5–52.0%, (62)] and buttermilk powder (46.5–49.0%; (61)). While lower ash content in NF-SBMP (6.38%) was found in comparison with RO-SBMP [7.48%, (53)], buttermilk powder [8.3–8.8%; (61)], SMP [8.2–8.6%, (62)], and yogurt powder [6.8–8%; (60)]. Demineralization of DSBM could explain the comparatively lower ash content of NF-SBMP than other dairy powders.

**Table 2:** Physico-chemical, reconstitution, functional, and anti-oxidative properties of Sour buttermilk powder by nanofiltration process (NF-SBMP) (Mean ± SD, *n* = 9 independent trials).

NF-SBMP	Parameters		NF-SBMP
Chemical composition and physical properties	TS (%)		98.04 ± 0.06
	Fat (%)		3.44 ± 0.12
	Protein (%)		49.04 ± 0.21
	Lactose (%)		38.35 ± 0.30
	Ash (%)		6.38 ± 0.16
	Free fat (% of total fat)		1.23 ± 0.13
	Hydroxymethylfurfural (μmol/Kg of powder)		1166.15 ± 1.17
	2-thiobarbituric acid, TBA (μg/mL)		0.10 ± 0.01
	Acidity (% LA)		1.31 ± 0.04
	pH (10% w/v solution), at 20°C		4.33 ± 0.01
	ζ-potential (mV) (1,000 × dilution)		−2.45 ± 0.33
	Water activity (a_w_)		0.22 ± 0.02
	Color values	L*	74.14 ± 0.15
		a*	2.17 ± 0.20
		b*	28.74 ± 0.03
Bulk and flow properties	Interstitial air content (cm^3^.100 g^−1^ powder)		78.73 ± 2.37
	Occluded air content (cm^3^.100 g^−1^ powder)		77.86 ± 1.15
	Loose bulk density (g.cm^−3^)		0.49 ± 0.02
	Packed bulk density (g.cm^−3^)		0.63 ± 0.01
	Particle density (g.cm^−3^)		1.25 ± 0.09
	Porosity (%)		60.60 ± 0.53
	Flowability (angle of repose, θ°)		32.10 ± 0.36
Reconstitution and functional properties	Wettability (s)		03.00 ± 0.00
	Dispersibility (%)		82.90 ± 0.83
	Solubility index (mL per 100 mL reconstituted)		77.50 ± 0.15
	Water binding capacity (g per g of protein)		4.20 ± 0.49
	Oil binding capacity (g per g of protein)		2.44 ± 0.38
	Foam capacity (%)		25.00 ± 1.72
	Foam stability (%)		18.02 ± 1.40
	Emulsion capacity (%)		31.35 ± 0.35
	Emulsion stability (%)		80.15 ± 0.44
	ƞ_Appa._(mPa.s, 10% w/v solution), at 100 s^−1^ and 20°C		5.14 ± 0.23
Particle size distribution	d_10_ μm		16.50 ± 0.10
	d_50_ μm		70.40 ± 0.26
	d_90_ μm		145.66 ± 1.15
	D_3,2_ μm		86.23 ± 1.04
	D_4,3_ μm		4.57 ± 0.01
	Span (%, dispersion index)		1.84 ± 0.02
	SSA (m^2^ kg^−1^)		131.1 ± 1.33

**Table 3:** Fatty acid composition in Sour buttermilk powder by nanofiltration process (NF-SBMP).

Types	Fatty acids	% total fatty acids
Short-chain fatty acids	Butyric acid (C4:0)	3.78
	Caproic acid (C6:0)	2.13
	Caprylic acid (C8:0)	0.81
	Capric acid (C10:0)	1.86
Medium-chain fatty acids	Lauric acid (C12:0)	2.35
	Myristic acid (C14:0)	12.44
	Myristoleic acid (C14:1) ^#^	0.67
Long-chain fatty acids	Pentadecylic acid (C15:0) *	1.15
	Palmitic acid (C16:0) *	36.07
	Hexadecenoic acid (C16:1)	1.69
	Margaric acid (C17:0) *	0.83
	Stearic acid (C18:0) *	12.43
	Oleic acid (C18:1) ^#^	1.08
	Linoleic acid (C18:2) ^#^	1.25
	Linolenic acid (C18:3) ^#^	0.12
	Arachidic acid (C20:0) *	0.59

*Saturated fatty acids; ^#^Unsaturated fatty acids.

Interaction between milk proteins and lactose occurs during heat treatment of milk and drying of milk concentrate, and is known as the Maillard reaction. It shows a detrimental loss of nutrition and deteriorates the color of milk products (63). The intensity of the Maillard reaction is indicated by HMF (64). NF-SBMP contained higher HMF (1166.15 μmol/Kg of powder) than RO-SBMP [1085.17 μmol/Kg of powder, (53)]. The degree of heat treatment, the protein-to-lactose ratio, and lower pH in concentrate collectively impacted the production of HMF in milk powder (65–67). High HMF content in NF-SBMP may be attributed to prolonged heat treatment of milk prior to fermentation. TBA value is a marker of lipid oxidation and a predictor of secondary oxidation product (e.g., carbonyls) generation (68). NF-SBMP shows in-line TBA value (Table 2) with RO-SBMP [0.15 μg/mL, (53)], and that can be attributed to the lower fat content in DSBM and CSBM, respectively.

The acidity of the reconstituted NF-SBMP solution (10%, w/v) (Table 2) was found to be lower than that of RO-SBMP [1.80%, LA; (53)]. Lower acidity of NF-SBMP could be attributed to the separation of lactic acid during the concentration of DSBM by the NF process. Similarly, pH of reconstituted NF-SBMP solution (10%, w/v) (Table 2) was also found higher than previous studies [4.22; (53)] while the same was lower than that of *lassi* powder [4.63–4.7, (59)] but lies within the range with different yogurt powders (4.3–5.3, (60)). Wade et al. (69) found that when pH drops, the *ζ* potential becomes less negative. Higher ζ potential in NF-SBMP (Table 2) over RO-SBMP [−0.18 mV, (53)] could be attributed to the gradual increase in pH of NF-SBMP due to the removal of lactic acid from DSBM. Lower (<0.25) a_w_ values guarantee powder stability during storage as well as simultaneously inhibit microbiological growth during storage (70). a_w_ of NF-SBMP (0.22) was similar to RO-SBMP [0.25, (53)], and it falls within the range with SMP and WMP [0.1–0.3; (71) and 0.23–0.303; (72)]. The color of milk powder plays a crucial role in consumer acceptance and applications toward product formulation. The L* and b* values of NF-SBMP (74.14 and 25.87) were slightly lower than those of RO-SBMP [74.69 and 26.62, (53)], while the a* value (2.17) was slightly higher than RO-SBMP [1.65; (53)]. The high HMF value of NF-SBMP could explain its brown color.

#### 3.2.2 Bulk, reconstitution, and functional properties of SBMP

IAC is the volume difference between the mass of the powder particles and the volume of the equal mass of packed or tapped powder, while OAC is the volume difference between the given mass of particles and the volume of air-free solids (73). IAC and OAC of NF-SBMP (Table 2) were found higher than RO-SBMP [74.62 and 8.41 cm^3^ 100 g^−1^ powder, (53)] but fall within the middle range milk powder [IAC_SMP & WMP_ 41–120 cm^3^.100 g^−1^, OAC_SMP & WMP_-10–200 cm^3^.100 g^−1^; (73)]. As per Schuck (73), IAC is affected by particle size distribution and degree of agglomeration, while OAC is affected by factors such as feed properties (i.e., air incorporation, whipping, foam stability), system used during spray drying, and processing conditions (i.e., type of atomizer). The d_90_ value in particle size distribution (Table 2) of NF-SBMP clearly indicates the degree of agglomeration in NF-SBMP, which explains its higher IAC value. Zang and Goff (74) reported that foaming increases with a rise in pH. Remarkably higher OAC value in NF-SBMP could be attributed to the higher pH (4.22) of CSBM than previous studies [3.22, (53)].

The bulk properties of food powders, such as loose and packed densities, porosity, and flowability, are substantially influenced by the particle size distribution (75). Powder having low bulk density requires more packaging volume. Here, slightly lower LBD and similar PBD and PD (Table 2) of NF-SBMP were found with RO-SBMP [0.53, 0.66, and 1.30 g.cm^−3^, (53)]. Here, a higher OAC of NF-SBMP could explain its lower LBD. Similarly, PD was also influenced by the presence of entrapped air. The NF-SBMP showed similar PBD and PD (Table 2) values with RO-SBMP (53). NF-SBMP showed slightly higher porosity over RO-SBMP [59.36%, (53)], but the same was markedly higher over yogurt powder [36.54%, (76)]. As per Fang et al. (77), powder porosity decreases with the increasing presence of smaller particles. Therefore, a lower value of smaller particles (Table 2) could explain the variation in porosity of NF-SBMP.

The ability of powder particles to flow without clumping or aggregating is known as flowability (73). Angle of repose is widely used to describe NF-SBMP, which has a *θ* value of 32.10, enabling it to be marked as a free-flowing powder, as Carr (78) reported that free-flowing powders have θ values up to 35°. Although the θ value of NF-SBMP was slightly higher than that of RO-SBMP [28.36°, (53)]. Milk powder having a larger particle diameter (>90 μm) possesses good flow behavior (73). NF-SBMP contained a d_90_ value of 145.66 μm (Table 2), which indicates the presence of large particles in NF-SBMP and advocates its free-flow property.

The time required for 1 g of powder sample to penetrate a still water surface is termed wettability. It is influenced by many variables, including surface charge and its activity, particle size, particle density, powder porosity, and the presence of moisture-absorbing components (73). Wettability of NF-SBMP was 3 s, and it was similar to that of RO-SBMP [3 s, (53)]. The range of wettability for SMP and WMP is < 15 s and 30–60 s, respectively. Furthermore, powder wetted in < 15 s is termed as “instant powder” (79). Phospholipids are found in SBM in good proportions [115.50 mg/100 g; (80)] as milk fat globule membrane disrupts during the churning process and migrates to the aqueous phase (81). Hence, NF-SBMP can be termed as “instant powder,” which could be due to the presence of phospholipids, a well-known surface-active agent.

The capacity of powder particles to evenly disperse in water under test conditions is known as dispersibility (73). Casein and whey protein interactions induced due to heat treatment have a significant impact on their functional properties and produce unstable dispersion (79). NF-SBMP shows higher dispersibility than RO-SBMP [73.74%, (53)]. Dairy powders such as SMP and WMP show dispersibility of ≥90% and ≥85%, respectively (82). The dispersibility of spray-dried yogurt powder was 351 s (76). As per Schokker et al. (83), dairy powders containing higher casein content exhibited poor dispersibility and took a longer time to disperse in water due to hydrophobic cross-linking interaction between casein micelles (84). Similarly, Maillard reaction products (methylglyoxal, glyoxal) develop hydrophobic cross-linking interactions with milk proteins (85). Higher protein and HMF content in NF-SBMP, and interaction between casein and whey proteins during heat treatment in SBM, may explain its intermediate dispersibility.

Powder solubility is considered a crucial feature for quality standards. It is usually determined using different methods (73). Solubility index of NF-SBMP was 77.50% that was found to be higher than RO-SBMP [71.50%, (53)] and yogurt powder [68.70%, (76)]. Although dairy powders (i.e., SMP and WMP) show a solubility index of >99% (73). Constituents such as lactose and salts in milk powder contribute hydrophilicity, and greater protein content exhibits hydrophobicity due to cross-linking interaction of casein micelles (84). Therefore, higher lactose content and lower protein may contribute to a higher solubility index in NF-SBMP over RO-SBMP.

It has been claimed that the way proteins interact with water determines the functional properties of food proteins. Numerous factors, such as protein concentration, lipids and salt content, temperature, pH, heat treatment intensity, hydrophilic polysaccharide content, and storage conditions, collectively affect the WBC of proteins (86). Furthermore, methods (drying or grinding) used to produce milk powders govern their particle size, topography, and porosity, which also influence WBC (87). WBC of NF-SBMP (Table 2) was slightly lower than RO-SBMP [4.34 g per g of protein (53)], but it was remarkably higher than whey powder [0.33–1.80 g per g of protein (53)]. Prolonged heat treatment resulted in whey protein denaturation and their attachment to casein micelles, which develops more polar sites for water adsorption (88, 89). It can be said that protracted exposure in heat treatment of milk and high protein content in NF-SBMP collectively contribute to higher WBC value. According to Wagner and Anon (90), solubility and WBC have an inverse relationship. In fact, a substantial amount of water becomes trapped in a stable protein matrix that is produced by denatured proteins. For increased WBC of NF-SBMP, a similar explanation can be applied.

The capacity to hold and absorb fat is known as OBC and is affected by particle size distribution. NF-SBMP showed in-line OBC (Table 2) with RO-SBMP [2.77 g per g of protein, (53)]. As per Zayas (86), mild and slow heat treatment of milk causes unfolding of whey proteins, which further increases hydrophobicity and oil-binding capacity. Therefore, the observed higher OBC of NF-SBMP may be explained by prolonged heat treatment of milk before fermentation.

The ability of a protein solution to retain air bubbles or foams at the protein and water interface is known as FC, while the ability of milk protein to stabilize foam lamella and entrap foam within the protein matrix is known as FS (91). Singh (92) reported that several factors, such as fat and protein content, preheat treatment, protein denaturation, and ionic strength, collectively affect foaming properties. Here, NF-SBMP showed higher foam capacity and stability (Table 2) than RO-SBMP [22.18 and 14.32%, (53)]. This could be attributed to higher pH, higher solubility index, and higher *ζ*-potential of NF-SBMP than RO-SBMP, as it has a direct correlation with the net proton charge.

EC is defined as an emulsifying property of a protein solution that retains oil at the oil–water interface. ES refers to the capacity of emulsion droplets to stay dispersed without clumping, aggregating, or whitening (86). Numerous factors, such as TS, protein, calcium content, pH, and particle size distribution, collectively influenced emulsification properties as per Meena et al. (93). Furthermore, according to Wong and Kitts (94), the degree of surface denaturation, protein solubility, lipid-to-protein ratio, and protein amphipathic character also collectively affect the emulsification properties of protein. Here, EC and ES of NF-SBMP (Table 2) were found similar with RO-SBMP [31.35 and 80.15%, (53)]. Lower solubility and naturally present components (phospholipid and MFGM in SBM) could be attributed to higher EC and ES in NF-SBMP.

Here, NF-SBMP showed a higher ƞ_Appa_ (Table 2) than RO-SBMP [4.09 mPa.s; (53)]. Sensorial attributes and consumer acceptance influenced rheological attributes in yogurt powder (95). As per Seth and coworkers (96), poor rheological properties of yogurt powder were attributed to its lower protein solubility. Hence, a higher solubility index of NF-SBMP can explain the higher ƞ_Appa_ of NF-SBMP over RO-SBMP.

#### 3.2.3 Particle size distribution in NF-SBMP

The particle size distribution of milk powder accounts for its appearance, flow characteristics, surface reactivity, and reconstitution quality. Factors such as feed characteristics, equipment used during processing, conditions maintained during processing, and drying collectively determine particle size (73). Particle size distribution (d_10_-16.50 μm_;_ d_50_-70.40 μm; d_90_-145.66 μm; D_4,3_–86.23 μm; D_3,2_–4.57 μm; span-1.84, SSA-131.1 m^2^ kg^−1^) of NF-SBMP indicates lower d_10_ and D_3,2_ values, while higher values of d_50_, d_90_, D_4,3_, span, and specific surface area (SSA) than RO-SBMP (53). Higher TS and viscosity of CSBM (Table 1) produced by NF over that produced by the RO process (53) may explain the observed fluctuation in particle size distributions of NF-SBMP. The SEM micrograph of NF-SBMP also supported the variation in particle size (Figure 3d).

#### 3.2.4 Fatty acid composition in NF-SBMP

Table 3 shows the fatty acid composition of NF-SBMP. Moderately lower concentration of palmitic acid (36.07%), followed by myristic acid (12.44%) and stearic acid (12.43%), was present in NF-SBMP than that of RO-SBMP [46.89, 15.88, and 13.47%, (53)]. Although similar fatty acid concentrations were found in cow milk powder [palmitic acid-33.80%, stearic acid-12.7%, (97)]. Application of higher churning temperature resulted in higher melting point fatty acids than low melting point in artisanal household method, and around 27% milk fat was lost during churning operation (19). Evidently, the melting points of the saturated fatty acids (palmitic, myristic, and stearic acids) were remarkably higher than those of unsaturated fatty acids, which include linoleic acid (1.25%), oleic acid (1.08%), and linolenic acid (0.12%).

#### 3.2.5 FTIR, XRD spectra, and SEM micrographs of NF-SBMP

FTIR is a very useful method for characterizing the organic components (solid, liquid, or gas) in a food system (98). Figure 3b shows the distinctive peaks of different chemical components of SBMP. Fat present in NF-SBMP shows higher absorption in 2,922–2,924, 2,852–2,854, 1,737–1,739 cm^−1^, while a lower C=O distinctive peak in 1,739–1,741 cm^−1^ and a drop in C-O frequency in 1168 indicate low fat concentration in NF-SBMP. Similarly, distinctive peaks for amide I and II in NF-SBMP were observed in 1,620 and 1,529 cm^−1^, respectively, which implies the presence of protein in NF-SBMP. While the presence of lactose in NF-SBMP was advocated via characteristic peaks in 800–1,250 cm^−1^ as per Lei et al. (99). These results were in-line with RO-SBMP (53).

One of the common and efficient analytical methods that has been used to determine the phase of crystalline materials is X-ray diffraction (XRD), and it is widely applied for the detection of lactose crystallization in milk powders (100). The identification of lactose crystals in SBMP was conducted and depicted in Figure 3c. The diffraction angle (2θ) of NF-SBMP (19.18°) was found slightly lower than RO-SBMP [20°; (53)]. Furthermore, peaks of *α*-lactose monohydrate, stable anhydrous α-lactose, and anhydrous α and *β*-lactose mixture (molar ratio of 5:3) were observed at diffraction angles of 19.1° (101), 20.0° (102), and 20.1° (103). Therefore, the 2θ value of NF-SBMP indicates that crystalline lactose is present in the sample, and it ultimately falls within the range (19.1–20.1°) that has been documented in scientific publications.

The presence of spherically-shaped, variable-sized powder particles was revealed by SEM micrographs of NF-SBMP (Figure 3d). Additionally, the particle size distribution of NF-SBMP (Table 2) supported the SEM micrographs that it contained the majority of low and medium-sized particles. Clustering in powder particles was observed due to amorphous lactose and fat particles that were also observed in RO-SBMP (53).

### 3.3 Utilization of NF permeate in the manufacturing of buffalo milk *chhana*

#### 3.3.1 Chemical composition and physical properties of chhana

Moisture content and yield of BMPC were significantly (*p* < 0.05) higher than those of other *chhana* samples (Table 4). This result indicates better water retention in BMPC over other *chhana* samples. Higher TS, fat loss in the whey of BMPC, and this explains the lowest dry matter of the BMPC since it has the highest moisture content. Bandyopadhyay et al. (56) described that a coagulant containing a lower concentration of acid produces a higher yield in *chhana*. The current result was also in line with the reported results by Bandyopadhyay et al. (56).

**Table 4:** Chemical composition, physical, and textural properties of different *chhana* and whey samples.

Parameters		Buffalo milk permeates treated *chhana* (BMPC)	Buffalo milk citric acid-treated *chhana* (BMCAC)	Cow milk citric acid-treated *chhana* (CMCAC)
Moisture (%)		61.50 ± 2.15^a^	44.13 ± 1.73^c^	49.16 ± 1.59^b^
Yield (%)		28.13 ± 0.24^a^	17.91 ± 0.44^b^	14.62 ± 0.71^c^
Color values	L*	81.52 ± 0.68^a^	79.18 ± 0.68^b^	77.31 ± 0.81^c^
	a*	−0.27 ± 0.06^a^	−0.38 ± 0.79^a^	−1.06 ± 0.05^b^
	b*	9.24 ± 0.62^c^	12.46 ± 0.78^b^	16.83 ± 0.24^a^
Instrumental texture profile analysis	Hardness (N)	07.22 ± 0.33^b^	20.46 ± 4.57^a^	10.73 ± 3.54^b^
	Adhesiveness (N.s)	−33.00 ± 6.75^a^	−19.11 ± 7.04^a^	−38.76 ± 13.61^a^
	Springiness	0.16 ± 0.00^ab^	0.18 ± 0.01^a^	0.14 ± 0.01^b^
	Cohesiveness	0.10 ± 0.01^b^	0.12 ± 0.01^ab^	0.13 ± 0.01^a^
	Gumminess	0.72 ± 0.02^b^	2.47 ± 0.39^a^	1.38 ± 0.33^b^
	Chewiness (N)	0.12 ± 0.00^b^	0.45 ± 0.10^a^	0.20 ± 0.06^b^
Whey streams	Total solids (%)	6.55 ± 0.62^a^	3.04 ± 0.73^b^	5.67 ± 0.10^a^
	Fat loss (%)	0.63 ± 0.05^a^	0.16 ± 0.01^b^	0.33 ± 0.06^b^
	pH	5.60 ± 0.04^a^	4.73 ± 0.23^b^	4.65 ± 0.03^b^
	Acidity (%LA)	0.53 ± 0.06^b^	1.96 ± 0.05^a^	1.80 ± 0.10^a^
	ƞ_Appa_, 100 s^−1^, mPa.s, at 20°C	2.40 ± 0.40^a^	1.95 ± 0.17^a^	1.98 ± 0.28^a^

Mean ± SD (*n* = 9 independent trails) with different superscripts ^abc^is significantly different (*p* < 0.05) with each other row-wise.

BMPC showed significantly (*p* < 0.05) higher L* value than the other two samples. The aggregation of casein micelles increased light scattering (104), and higher moisture content also increases light scattering of casein and makes the coagulum whiter (44). Higher yield of BMPC than other *chhana* samples directly correlates with its higher L* value. Similarly, significantly (*p* < 0.05) higher a* values of BMCAC and BMPC were observed, and the same may be attributed to the presence of biliverdin pigment in buffalo milk (43), while lower a* in CMCAC indicates its green color (44). Similarly, significantly (*p* < 0.05) higher b* value in CMCAC indicates the natural presence of carotenoids, while lower b* value in BMCAC and BMPC could be attributed to the absence of carotenoids as reported by Sindhu and Arora (43).

#### 3.3.2 Textural attributes of different chhana samples

Hardness of BMPC was significantly (*p* < 0.05) lower than that of other samples. Bandyopadhyay et al. (56) also reported similar findings during the preparation of *chhana* from citric acid (CA) and lactic acid (LA). Chang (105) reported that CA has a dissociation constant of 8.7 × 10^−4^ at 25 °C, while LA has 1.39 ×10^−4^ and this clearly indicates that LA is a weak acid as compared to CA. CA, when directly added to milk, causes a quick drop in pH. This rapid acidification immediately disrupts the stability of casein micelles. Citrates have a higher affinity for calcium ions and induce more calcium dissociation from the casein micelles. Furthermore, the rapid drop in pH causes proteins to lose water, resulting in a drier, harder curd. The curd structure was more rigid as the use of citric acid leads to a decrease in moisture retention and also results in lower yield. Meanwhile, LA slowly decreases the pH in milk, which allows for a more controlled acidification process, usually destabilizing casein micelles over time. It induces less calcium dissociation from casein micelles. This preserves some of the calcium-casein bonds, leading to a softer, more flexible curd. This process allows the casein micelles to retain more water, which results in a more hydrated, softer curd. The higher curd yield was attributed to its higher moisture content rather than higher TS recovery. The pKa of lactic acid is 3.85–3.86 at 25 °C (106, 107). The pKa of citric acid is 3.13, 4.76, and 6.4 (108, 109). This may explain the reason for the BMPC being softer. Significantly (*p* < 0.05) lower adhesiveness in BMPC was observed over BMCAC. The rate and degree to which a deformed material returns to its original condition following the removal of the deforming force is known as springiness (110), and the degree of *chhana* deformation before its rupture is known as cohesiveness. Springiness and cohesiveness of all the samples were statistically (*p* > 0.05) at par. Bandyopadhyay et al. (56) also reported that these two attributes of chhana were not affected by the concentration and type of coagulant used.

Gumminess is defined as the energy requirement for the breakdown of a food product into a ready-to-swallow state and is calculated via the multiplication of resultant hardness and cohesiveness (56). Significantly (*p* < 0.05) lower gumminess in BMPC was observed over BMCAC. Lower hardness value in BMPC may be attributed to lower gumminess, as it implies that less energy was required to produce the ready state for swallowing. Similarly, chewiness is the total energy during mastication of solid food into a ready-to-swallow state and is calculated by multiplying hardness, cohesiveness, and springiness (56, 111). Significantly (*p* < 0.05) lower chewiness was found in BMPC over BMCAC. The findings of this investigation are in line with the results published by Bandyopadhyay et al. (56), who reported that CA produces higher chewiness compared to LA in *chhana.*

## 4 Conclusion

Processing and preservation of SBM is challenging owing to its huge bulk with low TS, poor heat stability, and high acidity. Concentration of SBM by NF passed the majority of its water as permeate and markedly enhanced protein and lactose contents in the NF retentate. Subsequent spray drying of NF retentate resulted in sour buttermilk powder that contained protein as a major constituent. Upon characterization, this powder exhibited excellent wettability (instant solubility), flowability (*θ*), good WBC, FC, FS, and fatty acid properties along with intermediate dispersibility. NF permeate was used as a natural coagulant for the manufacture of chhana from buffalo milk. Notably, the obtained chhana was soft and in high demand for sweet-making.

Overall, this study established that SBM concentration by NF combined with spray drying is an effective approach for the sustainable valorization of this domestic waste. This process is simple, advocates zero waste generation, and can generate extra income for the farmers, along with environmental protection.

## Data Availability

The raw data supporting the conclusions of this article will be made available by the authors, without undue reservation.

## Glossary

NF: Nanofiltration
PBD: Packed or tapped bulk density
SBM: Sour buttermilk
HR: Hausner ratio
TS: Total solids
CI: Carr index
SBMP: Sour buttermilk powder
SI: Solubility index
SCBM: Sweet cream buttermilk
WBC: Water binding capacity
DSBM: Defatted sour buttermilk
OBC: Oil binding capacity
CSBM: Sour buttermilk concentrate
ƞAppa: Apparent viscosity
VRR: Volume reduction ratio
SSA: Specific surface area
CF: Concentration factor
XRD: X-ray diffractometer
FM: Flux mean
SMP: Skim milk powder
IF: Initial flux
WMP: Whole milk powder
FF: Final flux
HMF: Hydroxymethylfurfural
IAC: Interstitial air content
TBA: 2-thiobarbituric acid
OAC: Occluded air content
EC: Emulsifying capability
LBD: Loose bulk density
ES: Emulsion stability
RO: Reverse osmosis
BMPC: Buffalo milk permeates treated *chhana*
*Chhana*: Heat and acid coagulated milk gel
CMCAC: Cow milk citric acid-treated *chhana*
BMCAC: Buffalo milk citric acid-treated *chhana*
TA: Titratable acidity

## References

[1] Xue L Liu X Lu S Cheng G Hu Y Liu J . China’s food loss and waste embodies increasing environmental impacts. Nat Food. (2021) 2:519–28. doi: 10.1038/s43016-021-00317-6, 37117678

[2] Bellemare MF Çakir M Peterson HH Novak L Rudi J. On the measurement of food waste. Am J Agric Econ. (2017) 99:1148–58. doi: 10.1093/ajae/aax034

[3] Shafiee-Jood M Cai X. Reducing food loss and waste to enhance food security and environmental sustainability. Environ Sci Technol. (2016) 50:8432–43. doi: 10.1021/acs.est.6b01993, 27428555

[4] Kummu M de Moel H Porkka M Siebert S Varis O Ward PJ. Lost food, wasted resources: global food supply chain losses and their impacts on freshwater, cropland, and fertiliser use. Sci Total Environ. (2012) 438:477–89. doi: 10.1016/j.scitotenv.2012.08.092, 23032564

[5] Philippidis G Sartori M Ferrari E Barek R. Waste not, want not: a bio-economic impact assessment of household food waste reductions in the EU. Resour Conserv Recycl. (2019) 146:514–22. doi: 10.1016/j.resconrec.2019.04.016, 31274960 PMC6559263

[6] Buzby JC Hyman J. Total and per capita value of food loss in the United States. Food Policy. (2012) 37:561–70. doi: 10.1016/j.foodpol.2012.06.00

[7] Nahman A de Lange W Oelofse S Godfrey L. The costs of household food waste in South Africa. Waste Manag. (2012) 32:2147–53. doi: 10.1016/j.wasman.2012.04.012, 22608682

[8] Chen C Chaudhary A Mathys A. Nutritional and environmental losses embedded in global food waste. Resour Conserv Recycl. (2020) 160:104912. doi: 10.1016/j.resconrec.2020.104912.

[9] Cooper KA Quested TE Lanctuit H Zimmermann D Espinoza-Orias N Roulin A. Nutrition in the bin: a nutritional and environmental assessment of food wasted in the UK. Front Nutr. (2018) 5:19. doi: 10.3389/fnut.2018.00019, 29644218 PMC5882835

[10] Spiker ML Hiza HAB Siddiqi SM Neff RA. Wasted food, wasted nutrients: nutrient loss from wasted food in the United States and comparison to gaps in dietary intake. J Acad Nutr Diet. (2017) 117:1031–1040.e22. doi: 10.1016/j.jand.2017.03.015, 28522208

[11] Wang R Liu G Zhou L Yang Z Tang Z Lu S . Quantifying food loss along the animal products supply chain in China with large-scale field-survey based primary data. Resour Conserv Recycl. (2023) 188:106685. doi: 10.1016/j.resconrec.2022.106685, 38826717

[12] Amicarelli V Lagioia G Bux C. Global warming potential of food waste through the life cycle assessment: an analytical review. Environ Impact Assess Rev. (2021) 91:106677. doi: 10.1016/j.eiar.2021.106677, 38826717

[13] FAO. Food Wastage Footprint and Climate Change. (2014). Available online at: http://www.fao.org/nr/sustainability/food-loss-and-waste. (Accessed September 18, 2025).

[14] Usmani Z Sharma M Gaffey J Sharma M Dewhurst RJ Moreau B . Valorization of dairy waste and by-products through microbial bioprocesses. Bioresour Technol. (2022) 346:126444. doi: 10.1016/j.biortech.2021.126444, 34848333

[15] Mahboubi A Ferreira JA Taherzadeh MJ Lennartsson PR. Value-added products from dairy waste using edible fungi. Waste Manag. (2017) 59:518–25. doi: 10.1016/j.wasman.2016.11.017, 27864017

[16] Parashar A Jin Y Mason B Chae M Bressler DC. Incorporation of whey permeate, a dairy effluent, in ethanol fermentation to provide a zero waste solution for the dairy industry. J Dairy Sci. (2016) 99:1859–67. doi: 10.3168/jds.2015-10059, 26723112

[17] PIB. World Milk Day (June 01): Achievement of the Department of Animal Husbandry and Dairying (Ministry of Fisheries, Animal Husbandry and Dairying). (2024). Available online at: https://pib.gov.in/PressNoteDetails.aspx?NoteId=151889&ModuleId=3 (Accessed July 20, 2025)

[18] MOFPI. In Conversation Fermented Milk Special. PMFME E-Newsletter. (2025). Available online at: https://pmfme.mofpi.gov.in/pmfme/newsletters/enewsnovember1.html (Accessed August 1, 2025).

[19] Halder K Sahu JK Naik SN Mandal S Bag SK. Improvements in makkhan (traditional Indian cultured butter) production: a review. J Food Sci Technol. (2021) 58:1640–54. doi: 10.1007/s13197-020-04711-z, 33897003 PMC8021505

[20] Aneja RP Mathur BN Chandan CC Banerjee AK. "Fat-rich products". In: Technology of Indian Milk Products: Handbook on Process Technology Modernization for Professionals, Entrepreneurs and Scientists. New Delhi: Dairy India Yearbook (2002). p. 196.

[21] De S. "Indian dairy products". In: Outlines of Dairy Technology. New Delhi: Oxford University Press (2004). p. 463–4.

[22] Padghan PV Mann B Kumar R Sharma R Kumar A. Studies on bio functional activity of traditional lassi. Indian J Tradit Knowl. (2015) 1:124–31.

[23] Pal D Rajorhia GS. Buttermilk utilization in dairy industry. Indian Dairyman. (1985) 9:397–403.

[24] Meshram BD. Development of Long Life Soft Drink from Butter Milk. Karnal: National Dairy Research Institute (Deemed University) (1998).

[25] Brião VB Tavares CRG. Pore blocking mechanism for the recovery of milk solids from dairy wastewater by ultrafiltration. Braz J Chem Eng. (2012) 29:393–407. doi: 10.1590/s0104-66322012000200019

[26] Brião VB Tavares CRG. Scientific note: ultrafiltration of effluents from a dairy industry for nutrient recovery: effect of pressure and tangential velocity. Braz J Food Technol. (2012) 15:352–62. doi: 10.1590/s1981-67232012005000028

[27] Frappart M Jaffrin MY Ding LH Espina V. Effect of vibration frequency and membrane shear rate on nanofiltration of diluted milk, using a vibratory dynamic filtration system. Sep Purif Technol. (2008) 62:212–21. doi: 10.1016/j.seppur.2008.01.025

[28] Cholangi A Hossain MM. Separation of proteins and lactose from dairy wastewater. Chem Eng Process Process Intensif. (2006) 46:398–404. doi: 10.1016/j.cep.2006.05.022, 38826717

[29] Bortoluzzi AC Faitão JA Di Luccio M Dallago RM Steffens J Zabot GL . Dairy wastewater treatment using integrated membrane systems. J Environ Chem Eng. (2017) 5:4819–27. doi: 10.1016/j.jece.2017.09.018

[30] Suárez A Fidalgo T Riera FA. Recovery of dairy industry wastewaters by reverse osmosis. Production of boiler water. Sep Purif Technol. (2014) 133:204–11. doi: 10.1016/j.seppur.2014.06.041

[31] Kyrychuk I Zmievskii Y Myronchuk V. Treatment of dairy effluent model solutions by nanofiltration and reverse osmosis. Ukr Food J. (2014) 3:280–7.

[32] Laszlo Z Kertesz S Beszedes S Hovorka-Horvath Z Szabo G Hodur C. Effect of preozonation on the filterability of model dairy waste water in nanofiltration. Desalination. (2009) 240:170–9. doi: 10.1016/j.desal.2007.12.040

[33] Luo J Ding L. Influence of pH on treatment of dairy wastewater by nanofiltration using shear-enhanced filtration system. Desalination. (2011) 278:150–6. doi: 10.1016/j.desal.2011.05.025

[34] Rice GS Kentish SE O’Connor AJ Barber AR Pihlajamaki A Nystrom M . Analysis of separation and fouling behaviour during nanofiltration of dairy ultrafiltration permeates. Desalination. (2009) 236:23–9. doi: 10.1016/j.desal.2007.10.046

[35] Vourch M Balannec B Chaufer B Dorange G. Nanofiltration and reverse osmosis of model process waters from the dairy industry to produce water for reuse. Desalination. (2005) 172:245–56. doi: 10.1016/j.desal.2004.07.038

[36] Balannec B Vourch M Rabiller-Baudry M Chaufer B. Comparative study of different nanofiltration and reverse osmosis membranes for dairy effluent treatment by dead-end filtration. Sep Purif Technol. (2005) 42:195–200. doi: 10.1016/j.seppur.2004.07.013

[37] Li Y Shahbazi A Williams K Wan C. Separate and concentrate lactic acid using combination of nanofiltration and reverse osmosis membranes. Appl Biochem Biotechnol. (2008) 147:1–9. doi: 10.1007/s12010-007-8047-5, 18401749

[38] Bobrova AV Ostretsova NG. Prospects for use of nanofiltration buttermilk and whey concentrates in the technology of fermented milk products with an increased mass fraction of protein. IOP Conf Ser Earth Environ Sci. (2021) 624:012137. doi: 10.1088/1755-1315/624/1/012137

[39] Zscherpe C Weissgerber C Schwermann S. Development of a reverse osmosis and nanofiltration membrane cascade to produce skim milk concentrate. J Food Eng. (2023) 343:111376. doi: 10.1016/j.jfoodeng.2022.111376, 38826717

[40] Meyer P Hartinger M Sigler S Kulozik U. Concentration of milk and whey by membrane technologies in alternative cascade modes. Food Bioprocess Technol. (2017) 10:674–86. doi: 10.1007/s11947-016-1848-1

[41] Westergaard V. Milk Powder Technology: Evaporation and Spray Drying. Copenhagen, Denmark: NIRO A/S (1994).

[42] Chandan RC. "CHEESES soft and special varieties". In: Cabellero B Trugo LC Finglas PM, editors. Encyclopedia of Food Sciences and Nutrition. Amsterdam, Netherlands: Elsevier (2003). p. 1093–8.

[43] Sindhu JS Arora S. "Buffalo milk". In: Fuquay JW Fox PF McSweeney PLH, editors. Encyclopaedia of Dairy Sciences. San Diego: Academic Press (2011). p. 503–11.

[44] Chakraborty P Singh T Shivhare US Basu S. Understanding the effect of milk composition and milking season on quality characteristics of chhana. J Texture Stud. (2021) 52:45–56. doi: 10.1111/jtxs.12558, 32909288 PMC7891405

[45] St-Gelais D Haché S Gros-Louis M. Combined effects of temperature, acidification, and diafiltration on composition of skim milk retentate and permeate. J Dairy Sci. (1992) 75:1167–72. doi: 10.3168/jds.s0022-0302(92)77863-1

[46] AOAC Horwitz W. International Official Methods of Analysis. VA International Official Methods of Analysis Association of Official Analytical Chemist, vol. 2 Wilson Boulevard: Arlington, VA: Association of Official Analytical Chemists (AOAC). (1995).

[47] IS:4079. Method for Determination of Lactose by Lane Eyon. New Delhi: Bureau of Indian Standards (1967).

[48] Hall CW Hedrick TI. Drying of Milk and Milk Products. 2nd ed. New York, NY: Avi Publishing (1972).

[49] Keeney M Bassette R. Detection of intermediate compounds in the early stages of browning reaction in milk products. J Dairy Sci. (1959) 42:945–60. doi: 10.3168/jds.s0022-0302(59)90678-2

[50] Hegenauer J Saltman P Ludwig D Ripley L Bajo P. Effects of supplemental iron and copper on lipid oxidation in milk. 1. Comparison of metal complexes in emulsified and homogenized milk. J Agric Food Chem. (1979) 27:860–7. doi: 10.1021/jf60224a048, 512240

[51] IS: 11766. Method for Determination of Titratable Acidity in Milk Powder and Similar Products. New Delhi: Bureau of Indian Standards (1986).

[52] Mahadev GM Meena GS. Milk protein concentrates 80: does composition of buffalo milk matter for its poor functionality? Lebenson Wiss Technol. (2020) 131:109652. doi: 10.1016/j.lwt.2020.109652

[53] Manik S Meena GS Singh AK Khetra Y Singh R Arora S . Valorization of sour buttermilk (a potential waste stream): conversion to powder employing reverse osmosis and spray drying. Membranes. (2023) 13:799. doi: 10.3390/membranes13090799, 37755221 PMC10534478

[54] Chakraborty P Bhattacharya B Shivhare U Basu S. Investigation of heat-acid induced coagulation behaviour of whole milk systems employing front-face fluorescence spectroscopy. Int J Dairy Technol. (2020) 73:674–82. doi: 10.1111/1471-0307.12726

[55] BIS. Methods for Test for Dairy Industry - Chemical Analysis of Milk. Hand Book of Food Analysis. SP: 18 (Part XI - 1981). Dairy Products, 1st edn. New Delhi: Bureau of Indian Standards. (1981); 9:120–184.

[56] Bandyopadhyay M Chakraborty R Raychaudhuri U. The effect of coagulants on the texture of chhana (an acid and heat coagulated product made from milk). Int J Food Sci Technol. (2005) 40:799–810. doi: 10.1111/j.1365-2621.2005.00979.x

[57] Brião VB Vieira Salla AC Miorando T Hemkemeier M Cadore Favaretto DP. Water recovery from dairy rinse water by reverse osmosis: giving value to water and milk solids. Resour Conserv Recycl. (2019) 140:313–23. doi: 10.1016/j.resconrec.2018.10.007

[58] Suárez E Lobo A Alvarez S Riera FA Álvarez R. Demineralization of whey and milk ultrafiltration permeate by means of nanofiltration. Desalination. (2009) 241:272–80. doi: 10.1016/j.desal.2007.11.087

[59] Rawat K Kumari A Kumar R Ahlawat P Sindhu SC. Spray-dried lassi powder: process optimisation using RSM and physicochemical properties during storage at room and refrigerated temperature. Int Dairy J. (2022) 131:105374. doi: 10.1016/j.idairyj.2022.105374, 38826717

[60] Kumar P Mishra HN. Yoghurt powder—a review of process technology, storage and utilization. Food Bioprod Process. (2004) 82:133–42. doi: 10.1205/0960308041614918

[61] Thinkusadairy. Dry Buttermilk & Buttermilk Powder. Thinkusadairy.Org. (2023). Available online at: https://www.thinkusadairy.org/products/milk-powders/milk-powder-categories/dry-buttermilk-and-buttermilk-powder (Accessed August 1, 2025).

[62] Thinkusadairy. Non-Fat Dry Milk & Skim Milk Powder. Thinkusadairy.Org. (2023). Available online at: https://www.thinkusadairy.org/products/milk-powders/milk-powder-categories/non-fat-dry-milk-and-skim-milk-powder (Accessed August 1, 2024).

[63] Aalaei K Rayner M Tareke E Sjöholm I. Application of a dye-binding method for the determination of available lysine in skim milk powders. Food Chem. (2016) 196:815–20. doi: 10.1016/j.foodchem.2015.10.004, 26593559

[64] Delgado-Andrade C Seiquer I Haro A Castellano R Navarro MP. Development of the Maillard reaction in foods cooked by different techniques. Intake of Maillard-derived compounds. Food Chem. (2010) 122:145–53. doi: 10.1016/j.foodchem.2010.02.031

[65] Sert D Mercan E Aydemir S Civelek M. Effects of milk somatic cell counts on some physicochemical and functional characteristics of skim and whole milk powders. J Dairy Sci. (2016) 99:5254–64. doi: 10.3168/jds.2016-10860, 27179852

[66] Le TT Bhandari B Holland JW Deeth HC. Maillard reaction and protein cross-linking in relation to the solubility of milk powders. J Agric Food Chem. (2011) 59:12473–9. doi: 10.1021/jf203460z, 22007925

[67] Baldwin AJ Ackland JD. Effect of preheat treatment and storage on the properties of whole milk powder-changes in physical and chemical properties. Neth Milk Dairy J. (1991) 45:169–81.

[68] Stapelfeldt H Bjørn H Skovgaard IM Skibsted LH Bertelsen G. Warmed-over flavour in cooked sliced beef chemical analysis in relation to sensory evaluation. Z Lebensm Unters Forsch. (1992) 195:203–8. doi: 10.1007/bf01202796

[69] Wade T Beattie JK Rowlands WN Augustin M-A. Electroacoustic determination of size and zeta potential of casein micelles in skim milk. J Dairy Res. (1996) 63:387–404. doi: 10.1017/s0022029900031915

[70] Koc B Yilmazer MS Balkır P Ertekin FK. Spray drying of yogurt: optimization of process conditions for improving viability and other quality attributes. Dry Technol. (2010) 28:495–507. doi: 10.1080/07373931003613809

[71] Szulc K Lenart A. Effect of composition on physical properties of food powders. Int Agrophys. (2016) 30:237–43. doi: 10.1515/intag-2015-0084

[72] Pugliese A Cabassi G Chiavaro E Paciulli M Carini E Mucchetti G. Physical characterization of whole and skim dried milk powders. J Food Sci Technol. (2017) 54:3433–42. doi: 10.1007/s13197-017-2795-1, 29051638 PMC5629152

[73] Schuck P. "Milk powder: physical and functional properties of Milk powders". In: Fuquay JW Fox JW Mcsweeney PF, editors. Encyclopedia of Dairy Sciences. United Kingdom: Elsevier. (2011). p. 117–22.

[74] Zhang Z Goff HD. Protein distribution at air interfaces in dairy foams and ice cream as affected by casein dissociation and emulsifiers. Int Dairy J. (2004) 14:647–57. doi: 10.1016/j.idairyj.2003.12.007

[75] Barbosa-Cánovas GV Ortega-Rivas E Juliano P Yan H. Food Powders: Physical Properties, Processing, and Functionality, vol. 86 New York, NY: Kluwer Academic/Plenum Publishers (2005).

[76] Koç B Sakin-Yılmazer M Kaymak-Ertekin F Balkır P. Physical properties of yoghurt powder produced by spray drying. J Food Sci Technol. (2014) 51:1377–83. doi: 10.1007/s13197-012-0653-8, 24966433 PMC4062699

[77] Fang Y Selomulya C Chen XD. On measurement of food powder reconstitution properties. Dry Technol. (2007) 26:3–14. doi: 10.1080/07373930701780928

[78] Carr RL. Evaluating flow properties of solids. Chem Eng. (1965) 18:163–8.

[79] Kelly J Kelly PM Harrington D. Influence of processing variables on the physicochemical properties of spray dried fat-based milk powders. L Lait. (2002) 82:401–12. doi: 10.1051/lait:2002019

[80] Pimentel L Gomes A Pintado M Rodríguez-Alcalá LM. Isolation and analysis of phospholipids in dairy foods. J Anal Methods Chem. (2016) 2016:1–12. doi: 10.1155/2016/9827369, 27610267 PMC5005530

[81] Corredig M Roesch RR Dalgleish DG. Production of a novel ingredient from buttermilk. J Dairy Sci. (2003) 86:2744–50. doi: 10.3168/jds.s0022-0302(03)73870-3, 14507009

[82] Tamime A. In: Tamime A, editor. Dairy Fats and Related Products. Chichester, West Sussex, United Kingdom: Wiley-Blackwell (2009)

[83] Schokker EP Church JS Mata JP Gilbert EP Puvanenthiran A Udabage P. Reconstitution properties of micellar casein powder: effects of composition and storage. Int Dairy J. (2011) 21:877–86. doi: 10.1016/j.idairyj.2011.05.004

[84] McSweeney DJ Maidannyk V Montgomery S O’Mahony JA McCarthy NA. The influence of composition and manufacturing approach on the physical and rehydration properties of milk protein concentrate powders. Foods. (2020) 9:236. doi: 10.3390/foods9020236, 32098298 PMC7074018

[85] Le TT Bhandari B Deeth HC. Chemical and physical changes in milk protein concentrate (MPC80) powder during storage. J Agric Food Chem. (2011) 59:5465–73. doi: 10.1021/jf2003464, 21539356

[86] Zayas JF Functionality of proteins in food. 228–259. Berlin, Heidelberg: Springer-Verlag Berlin (1997).

[87] Knightbridge J. P. Goldman A. Water absorptive capacity of dried milk products. N Z J Dairy Sci Technol (1975);10:152–157.

[88] Li H Zhao T Li H Yu J. Effect of heat treatment on the property, structure, and aggregation of skim milk proteins. Front Nutr. (2021) 8:714869. doi: 10.3389/fnut.2021.714869, 34604276 PMC8485980

[89] Heldman DR Hall CW Hedrick TI. Vapor equilibrium relationships of dry milk. J Dairy Sci. (1965) 48:845–52. doi: 10.3168/jds.s0022-0302(65)88349-7, 14330745

[90] Wagner JR Añon MC. Influence of denaturation, hydrophobicity and sulfhydryl content on solubility and water absorbing capacity of soy protein isolates. J Food Sci. (1990) 55:765–70. doi: 10.1111/j.1365-2621.1990.tb05225.x

[91] Ho TM Bhandari BR Bansal N. Functionality of bovine milk proteins and other factors in foaming properties of milk: a review. Crit Rev Food Sci Nutr. (2022) 62:4800–20. doi: 10.1080/10408398.2021.1879002 33527840

[92] Singh H. "Functional properties of milk proteins". In: Fuquay JW Fox JW Mcsweeney PF, editors. Encyclopedia of Dairy Sciences. United Kingdom: Elsevier. (2011). p. 887–93.

[93] Meena GS Singh AK Panjagari NR Arora S. Milk protein concentrates: opportunities and challenges. J Food Sci Technol. (2017) 54:3010–24. doi: 10.1007/s13197-017-2796-0, 28974785 PMC5603002

[94] Wong PYY Kitts DD. A comparison of the buttermilk solids functional properties to nonfat dried milk, soy protein isolate, dried egg white, and egg yolk powders. J Dairy Sci. (2003) 86:746–54. doi: 10.3168/jds.s0022-0302(03)73655-8, 12703609

[95] Sakin-Yilmazer M Koç B Balkir P Kaymak-Ertekin F. Rheological behavior of reconstituted yoghurt powder—an optimization study. Powder Technol. (2014) 266:433–9. doi: 10.1016/j.powtec.2014.06.060

[96] Seth D Mishra HN Deka SC. Effect of spray drying process conditions on bacteria survival and acetaldehyde retention in sweetened yoghurt powder: an optimization study. J Food Process Eng. (2017) 40, 1–10. doi: 10.1111/jfpe.12487

[97] Marconi E Panfili G. Chemical composition and nutritional properties of commercial products of mare milk powder. J Food Compos Anal. (1998) 11:178–87. doi: 10.1006/jfca.1998.0573

[98] Song K. "Micro-and Nano-fillers used in the rubber industry". In: Thomas S Hanna JM, editors. Progress in Rubber Nanocomposites. Sawston, UK: Woodhead Publishing (2017). p. 41–80.

[99] Lei Y Zhou Q Zhang Y-L Chen J-B Sun S-Q Noda I. Analysis of crystallized lactose in milk powder by Fourier-transform infrared spectroscopy combined with two-dimensional correlation infrared spectroscopy. J Mol Struct. (2010) 974:88–93. doi: 10.1016/j.molstruc.2009.12.030

[100] Bunaciu AA Udriştioiu EG Aboul-Enein HY. X-ray diffraction: instrumentation and applications. Crit Rev Anal Chem. (2015) 45:289–99. doi: 10.1080/10408347.2014.949616, 25831472

[101] Fries DC Rao ST Sundaralingam M. Structural chemistry of carbohydrates. III. Crystal and molecular structure of 4-O-β-D-galactopyranosyl-α-D-glucopyranose monohydrate (α-lactose monohydrate). Acta Crystallogr B. (1971) 27:994–1005. doi: 10.1107/s0567740871003364

[102] Buma TJ Wiegers GA. X-ray powder patterns of lactose and unit cell dimensions of β-lactose. Neth Milk Dairy J. (1967) 21:208–10.

[103] Simpson TD Parrish FW Nelson ML. Crystalline forms of lactose produced in acidic alcoholic media. J Food Sci. (1982) 47:1948–51. doi: 10.1111/j.1365-2621.1982.tb12920.x

[104] Ahmad S Gaucher I Rousseau F Beaucher E Piot M Grongnet JF . Effects of acidification on physico-chemical characteristics of buffalo milk: a comparison with cow's milk. Food Chem. (2008) 106:11–7. doi: 10.1016/j.foodchem.2007.04.021.

[105] Chang R. Physical Chemistry with Applications to Biological Systems. 2nd ed. London: Macmillan (1981).

[106] Wang C Chang T Yang H Cui M. Surface physiological changes induced by lactic acid on pathogens in consideration of pKa and pH. Food Control. (2014) 46:525–31. doi: 10.1016/j.foodcont.2014.06.024

[107] Resch JJ Daubert CR Foegeding EA. The effects of acidulant type on the rheological properties of beta-lactoglobulin gels and powders derived from these gels. Food Hydrocoll. (2005) 19:851–60. doi: 10.1016/j.foodhyd.2004.10.034

[108] Rooben R Raveendran S Palanisamy AB Ashok P Mukesh Kumar A Parameswaran B. Insight into citric acid: a versatile organic acid. Fuel. (2022) 327:125181. doi: 10.1016/j.fuel.2022.125181

[109] Qiao H Cui J Ouyang S Shi J Ouyang J. Comparison of dilute organic acid pretreatment and a comprehensive exploration of citric acid pretreatment on corn cob. J Renew Mater. (2019) 7:1197–207. doi: 10.32604/jrm.2019.07735

[110] Civille GV Szczesniak AS. Guidelines to training a texture profile panel. J Texture Stud. (1973) 4:204–23. doi: 10.1111/j.1745-4603.1973.tb00665.x

[111] Yang CST Taranto MV. Textural properties of mozzarella cheese analogs manufactured from soyabeans. J Food Sci. (1982) 47:906–10. doi: 10.1111/j.1365-2621.1982.tb12742.x

